# 3D printing of surgical hernia meshes impregnated with contrast agents: in vitro proof of concept with imaging characteristics on computed tomography

**DOI:** 10.1186/s41205-018-0037-4

**Published:** 2018-12-07

**Authors:** David H. Ballard, Udayabhanu Jammalamadaka, Karthik Tappa, Jeffery A. Weisman, Christen J. Boyer, Jonathan Steven Alexander, Pamela K. Woodard

**Affiliations:** 1 Mallinckrodt Institute of Radiology, Washington University School of Medicine, 510 S. Kingshighway Blvd, Campus Box 8131, St. Louis, MO 63110 USA; 2 Molecular and Cellular Physiology, Louisiana State University Health Sciences Center, Shreveport, LA USA

**Keywords:** 3D printing, Personalized medicine, Additive manufacturing, Imaging, Radiology, Medical devices

## Abstract

**Background:**

Selected medical implants and other 3D printed constructs could potentially benefit from the ability to incorporate contrast agents into their structure. The purpose of the present study is to create 3D printed surgical meshes impregnated with iodinated, gadolinium, and barium contrast agents and characterize their computed tomography (CT) imaging characteristics. Commercial fused deposition layering 3D printing was used to construct surgical meshes impregnated with imaging contrast agents in an in vitro model. Polycaprolactone (PCL) meshes were printed containing iodinated, gadolinium, or barium contrast; control PCL meshes without contrast were also fabricated. The three different contrast agents were mixed with PCL powder and directly loaded into the 3D printer. CT images of the three contrast-containing meshes and the control meshes were acquired and analyzed using small elliptical regions of interest to record the Hounsfield units (HU) of each mesh. Subsequently, to test their solubility and sustainability, the contrast-containing meshes were placed in a 37 °C agar solution for 7 days and imaged by CT at days 1, 3 and 7.

**Results:**

All 3D printed meshes were visible on CT. Iodinated contrast meshes had the highest attenuation (2528 mean HU), significantly higher than both and gadolinium (1178 mean HU) and barium (592 mean HU) containing meshes. Only barium meshes sustained their visibility in the agar solution; the iodine and gadolinium meshes were poorly perceptible and had significantly lower mean HU compared to their pre-agar solution imaging, with iodine and gadolinium present in the adjacent agar at day 7 CT.

**Conclusion:**

3D prints embedded with contrast materials through this method displayed excellent visibility on CT; however, only barium mesh maintained visibility after 7 days incubation on agar at human body temperature. This method of 3D printing with barium may have potential applications in a variety of highly personalized and CT visible medical devices.

## Background

Three-dimensional (3D) printing has had progressively more uses in medicine, expanding from anatomic models and surgical guides to implants and imaging phantoms [1]. Bioactive 3D printing has been used to impregnate drugs, hormones, and other substances into models, instruments, and implants, including surgical meshes [1–5]. Iodine has been successfully incorporated into 3D printed constructs and imaged with CT [6].

Materials that can increase the x-ray attenuation of CT broadly include substances used for oral and intravenous contrast in CT and fluoroscopic examinations, e.g., barium sulfate and iodine. Commercial contrast agents are not used in clinical practice of coating meshes due to inherent toxicities, short half-lives, and solubility of these materials in intra-abdominal compartments [7–9]. 3D printing technologies have the capability to incorporate contrast materials within the structure of surgical meshes while leaving other materials on the outside. In other words, the inner contrast-containing material is surrounded/shielded by a non-toxic material that lacks contrast and is likely impermeable to the contrast agent.

A potential application of 3D printing with contrast agents, such as the present simple proof-of-concept study, is to construct custom hernia meshes. Over 1 million hernia repairs are performed annually in the United States, the majority of which are inguinal hernias (approximately 800,000) [10] with approximately 350,000 ventral/incisional hernia operations [11]. Ventral/incisional and inguinal hernia recurrence following mesh repair ranges from 15 to 32% for ventral/incisional hernias [12–14] to 0.5–10% with inguinal hernias [15, 16]. Recurrent hernias are often predictable based on patient symptoms and confirmed by physical examination. Computed tomography (CT) and magnetic resonance (MR) imaging are used to diagnose suspected hernia recurrence or secondary complications in patients following mesh repair, particularly in ventral/incisional hernia repair [17]. In clinical practice, these meshes have variable visibility on CT [18] and MR imaging [19–22]. 3D printing may allow for highly visible medical devices with patient-specific geometries. The purpose of our current study was to create 3D printed meshes impregnated with barium-, iodinated-, and gadolinium-containing contrast agents and characterize their CT attenuation characteristics both after printing and then when kept at human body temperature over the course of 7 days. Moreover, this study serves as a proof of concept for other 3D printed medical implants as well as surgical devices requiring radio-opacity that may benefit from increased CT visibility by the addition of contrast agents.

## Methods

Commercial fused deposition layering 3D printing was used to create surgical meshes infused with imaging contrast agents. Computer-aided design files were generated in the shape of surgical meshes. These designs were manufactured using a Hyrel System 30 M 3D printer (Hyrel 3D, Norcross, GA). Three different contrast agents were used to impregnate the mesh structure including barium (barium sulfate powder; Sigma-Aldrich, St. Louis, MO), iodinated contrast (Optiray 350 [loversol], Mallinckrodt Inc., St. Louis, MO), and a gadolinium-based contrast medium (Dotarem [gadoterate meglumine]; Guerbet LLC, Bloomington, IN); control meshes without these contrast additives were also fabricated. The two commercial intravenous contrast agents (Optiray 350 and Dotarem) were selected based on the convenience and availability of these agents, which are commonly used.

Fused deposition modeling 3D printing with the Hyrel printer was performed using a KRA 15 print head, which directly prints using emulsified materials loaded into the print head rather than filaments, eliminating the need for an intermediate filament extrusion step. For 3D printing control meshes without contrast, polycaprolactone (PCL) powder was loaded directly into the print head. For 3D printing contrast-impregnated meshes, PCL powder mixed with contrast agents was also loaded directly into the print head. Mixing contrast agents with PCL was performed in one of two ways for barium powder or liquid iodinated and gadolinium contrast. For barium powder, calculated amounts of contrasting agent and PCL powder (1:10 wt/wt) were hand mixed thoroughly in a mortar and pestle under a fume hood. Iodinated and gadolinium contrast agents were also mixed with PCL powder in this manner; however, since these two commercial agents are in liquid form, the mixtures were allowed to air-dry in a chemical fume hood for 72 h. For printing the three contrast-containing meshes along with the control PCL mesh, 10 g of the three contrast-containing dried mixtures along with bland PCL powder were loaded into the KRA 15 print head for Hyrel printer. To 3D print the mesh, the print head temperature was maintained around 125-130C at a speed of 7 mm/s and at layer height 0.2 mm.

For CT imaging, 2 meshes impregnated with iodine and barium, 1 mesh impregnated with gadolinium, and 2 PCL control meshes were arranged 1 cm apart, and images were acquired with a kVp of 120, mAs of 220, using a slice thickness of 0.6 mm. CT imaging was performed using a Siemens Biograph 40 PET/CT scanner [Siemens, Munich, Germany]). CT images were analyzed using Vitrea Enterprise Suite (version 6.7, Vital Images, Inc., Minnetonka, Minnesota, USA) using a small elliptical region of interest (ROI) to acquire the mean Hounsfield units (HU) of each mesh. Fifteen mean HU were acquired for each of the four meshes.

To test the stability of the radio-opacity of the 3D printed mesh constructs in a solution at body temperature, each contrast-infused mesh, as well as the control PCL mesh (3 of each type and 3 controls, *n* = 12), were incubated in agar for 7 days at 37 °C. Cell culture grade agar (Millipore Sigma, MO) was dissolved in deionized water and sterilized. Molten agar was poured into 60 mm petri dishes. 3D printed mesh constructs of 2x2cm dimensions were placed in petri dishes after the agar solidified. Extra molten agar was poured into the petri dishes after placing the mesh to ensure complete immersion of mesh in agar media. After complete gelation of agar, all the petri dishes including control agar were placed in an incubator at 37 °C. Petri dishes were imaged with CT using the same imaging parameters previously mentioned on day 1, day 3 and day 7 after implantation into the 37 °C agar environment. CT images of the agar implanted mesh fragments were analyzed in a similar manner as previously described with Vitrea Enterprise Suite. A small elliptical ROI was placed on the mesh itself and a large circular ROI was placed on the background agar for days 1, 3, and 7.

Differences between mean HU values for each of the four meshes as well as the mesh in the agar solution were compared using one-way analysis of variance. A *p* value of < 0.05 was considered statistically significant.

## Results

**Fig. 1:**
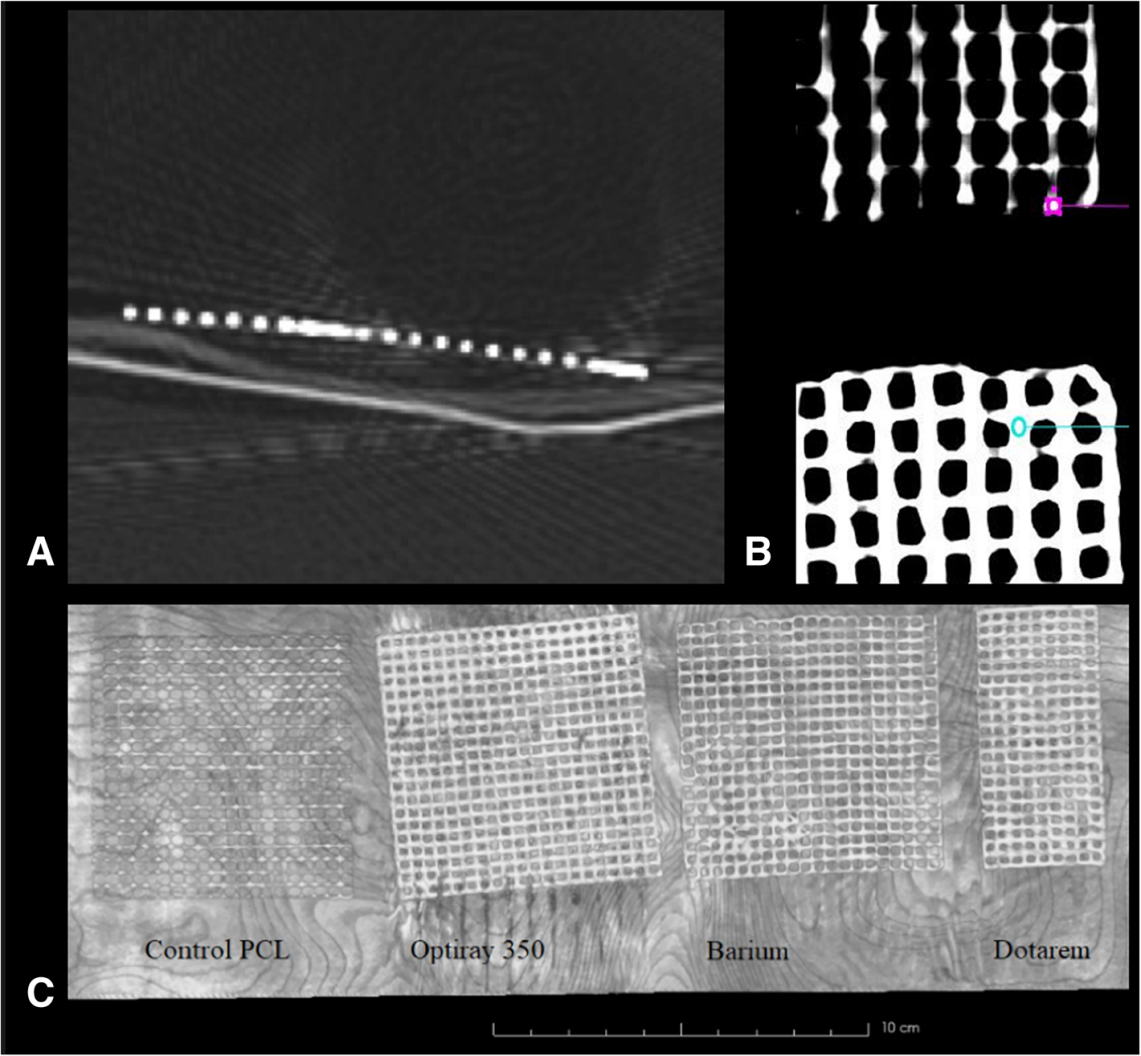
**a** Source CT image of iodinated contrast containing 3D printed mesh. **b** Coronal reconstructions depicting the small regions of interests used to analyze the mean Hounsfield units for each mesh. The barium mesh (top; purple region of interest) and iodinated contrast mesh (bottom; blue region of interest) are illustrated. **c** Maximum intensity project coronal reconstruction (**c**) of the three different contrast-impregnated polycaprolactone 3D printed meshes along with the control polycaprolactone 3D printed mesh

All contrast-containing 3D printed meshes were visible on CT (Fig. 1), each showing mean attenuation greater than 500 HU. Iodinated contrast displayed a mean HU of 2529 + 426, gadolinium contrast displayed mean HU of 1178 + 259, barium displayed mean HU of 592 + 186, and control 3D prints displayed mean HU of − 378 + 122 (Table 1). The iodinated contrast-containing 3D printed mesh had significantly higher attenuation values compared to all other meshes and gadolinium contrast-containing 3D printed mesh had significantly higher attenuation values compared to the barium and the control mesh (Table 1).

**Table 1:** Mean Hounsfield units of the three contrast-containing mesh-types in comparison to each other and the control meshes

3D Printed Meshes	Mean HU (± SD)	Significantly higher attenuation compared to other meshes	*p*-value
Iodinated contrast-containing (*n* = 2)	2529 ± 426	Greater than gadolinium, barium, and control	< 0.0001, < 0.0001, < 0.0001
Gadolinium contrast-containing (n = 1)	1178 ± 259	Greater than barium and control	< 0.0001, < 0.0001
Barium (*n* = 2)	592 ± 186	Greater than control	< 0.0001
Control (n = 2)	− 378 ± 122	None	Not applicable

*HU* Hounsfield units

*SD* Standard deviation

**Fig. 2:**
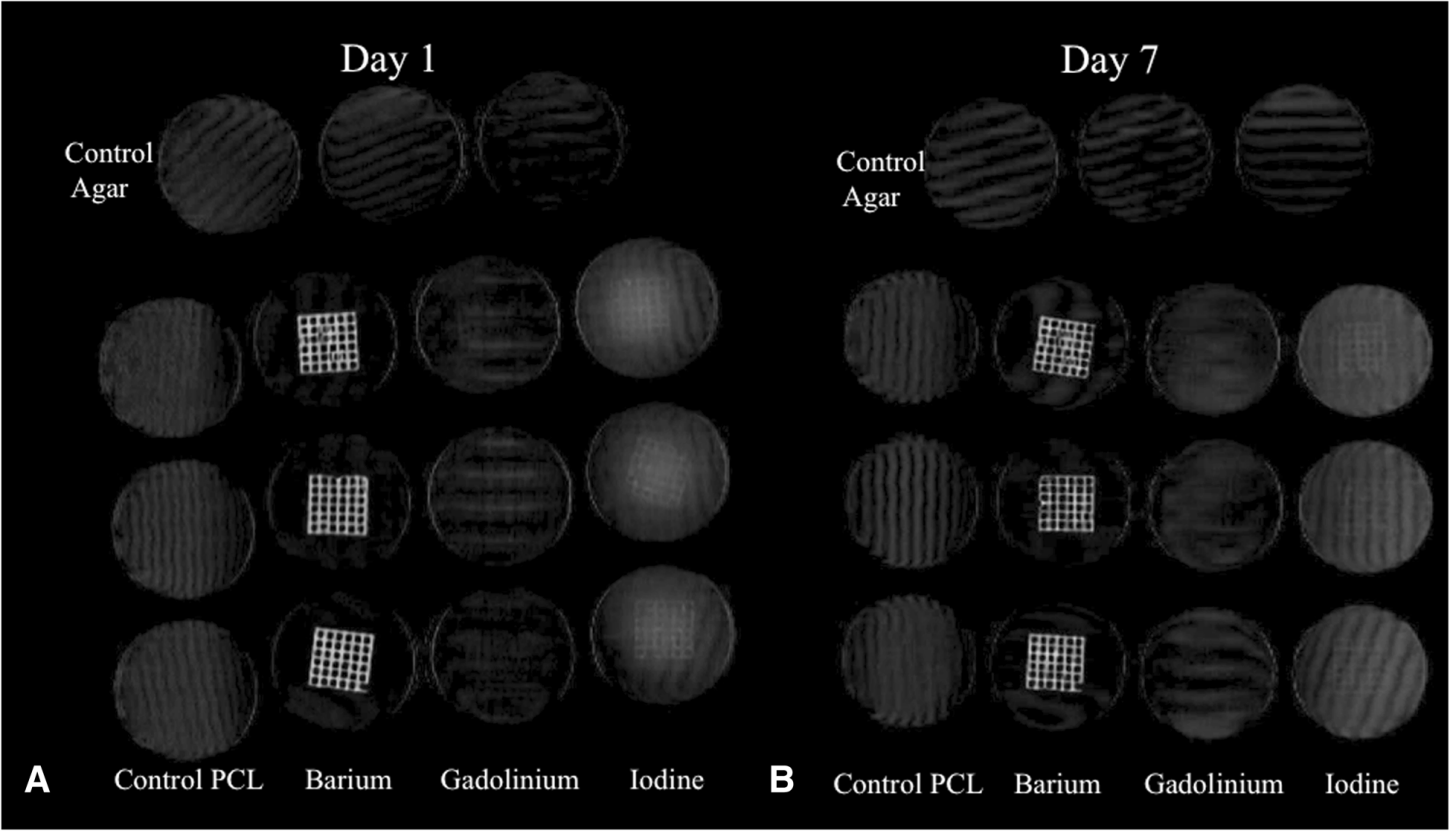
Coronal volume rendering of the contrast impregnated and control meshes in the 37 °C. agar solution at day 1 (**a**) and day 7 (**b**). Note the visibility of the barium mesh sustained at both time periods, poor perceptibility of the gadolinium meshes at either time period, and that more iodine is present in the surrounding agar at day 7 (**b**) compared to day 1 (**a**)

In the agar solution at simulated body temperature, the barium mesh was easily visible for all time periods, the iodine mesh was most perceptible at day 1 and less at day 7, and the gadolinium mesh was poorly perceptible at all time periods (Fig. 2). Objectively, the composite mean HU for the barium mesh in the agar solution was not significantly different compared to the pre-agar imaging (agar solution barium mesh = 541 + 133 mean HU vs pre-agar = 592 + 186 mean HU, *p* = 0.48) whereas both the agar solution iodine and gadolinium meshes had significantly lower mean HU compared to pre-agar imaging (agar solution iodine mesh = 194 + 54 mean HU vs pre-agar = 2529 + 426 mean HU, *p* < 0.001; agar solution gadolinium mesh = 44 + 19 mean HU vs pre-agar = 1178 + 259 mean HU, p < 0.001), with apparent contrast-agent visibility in the adjacent agar.

There were no significant differences within any of the same contrast meshes or their background agar at subsequent CT acquisitions at day 1, day 3, or day 7. The iodine meshes did visually become less perceptible from day 1 to day 7 (Fig. 2). The mean HU for the iodine meshes decreased upon repeat CT measurements (mean HU day 1 = 247, mean HU day 3 = 177, mean HU day 7 = 158), but again, this did not achieve statistical significance (*p* = 0.39–1.00).

## Discussion

In the present study, we describe fused deposition layering 3D printing as a process for impregnating contrast materials into 3D printed objects, with the proof-of-concept focusing on surgical mesh. Excellent visibility was demonstrated for CT imaging using all three contrast agents; however, contrast stability over time was demonstrated only with the barium infused mesh. The fused deposition layering 3D printing process described in the present study can potentially be applied for developing medical implants, with contrast in all layers – or all but the most external layers, for additional contrast-material containment. Moreover, contrast-impregnated fused deposition layering 3D printing can be used to create anatomic models to be CT scanned for pre-procedural planning, for image-guided therapies, or as phantoms.

In the simulated tissue environment (agar solution at 37 °C), only barium retained its visibility with a consistent mean HU compared to the pre-agar solution. The attenuation of both iodine and gadolinium both significantly decreased and were poorly visually perceptible in the agar solution, with apparent increase in contrast-material within the agar suggesting leaching of the material into the agar at body temperature.

Surgical meshes have variable appearances on imaging. On CT and MR imaging, different commercial meshes are either not visible, indirectly or poorly visible, or highly-visible. For CT, meshes with intrinsic high attenuation will have better visibility on CT [18]. There have been a number of “MR visible” meshes that design the mesh to accentuate signal voids. These meshes do not produce signal, but rather accentuate their signal voids by incorporating materials such as iron particles to achieve visibility by sharp signal dropout compared to surrounding tissue [19, 23].

In this study, we describe a novel method of incorporating contrast materials into 3D printed constructs using a fused-deposition modeling 3D printer. The incorporation of the contrast into the 3D printed construct itself increases the volume of contrast that can be incorporated compared to superficially coating with contrast and “protects” the contrast material deep in the construct from dilution that may occur with handling or cleaning/sterilization procedures. Although this technique may or may not have future use in printing custom meshes, there are a number of immediate ways this contrast-incorporating technique can be potentially used. Broadly, potential applications where impregnated contrast materials may be helpful include 3D printed phantoms, anatomic models, procedural/surgical instruments, and implants other than surgical mesh. 3D printed constructs have been used for a number of CT phantom studies [24–27]. The contrast-incorporating technique described in the present study may be used to increase x-ray attenuation of anatomic structures, such as high proportions of contrast being used to delineate high-density structures such as the axial and appendicular skeleton or at a diluted concentration to distinguish intermediate-density structures such as visceral organs from surrounding fat. Another potential application is printing anatomic models for simulation and training of fluoroscopic or CT-guided procedures. Few studies have reported 3D printed constructs being used to facilitate CT or fluoroscopic procedures [28, 29].

There are a number of limitations to this study. A single base material, PCL, was tested using a single commercial fused deposition modeling 3D printer. PCL was chosen given its ease to work with, availability, and that it has been previously used in studies impregnating drugs and other bioactive materials into 3D printed constructs [2–4]. The specific commercial print head used in this study directly used the contrast-PCL mixtures to print the meshes, without an intermediate filament extrusion step. Future studies to validate the method of contrast impregnated 3D printed constructs could include broadening the number of materials used, including common materials such as polylactic acid and polyvinyl alcohol, and incorporating other commercial 3D printers that require a preceding filament extrusion step. The 3D printed meshes in this study were imaged outside of tissues and in isolation; the appearance of a surgically implanted mesh may vary dramatically when embedded in tissues. The nature of this project was to demonstrate utility of this synthetic approach in creating materials with ideal imaging properties. Highly visible commercial meshes used in clinical practice are chosen for the factors of the mesh with relevance to ease of insertion and proven efficacy. At the time of this writing, 3D printed meshes have not been reported in humans; however, a number of in vitro studies have demonstrated the feasibility of surgical meshes impregnated with drugs or hormones [3, 4]. To fully validate the use of these mesh types in patients will require pre-clinical animal studies that compare the surgical efficacy toxicity, imaging characteristics, and longevity of imaging characteristics.

## Conclusion

This study describes a novel method to incorporate contrast materials into 3D printed constructs using a commercial fused deposition modeling printer. PCL was used as the base material along with barium powder and commercial liquid iodinated and gadolinium intravenous contrast agents. 3D printed meshes infused with contrast materials were highly visible on CT, with mesh impregnated with barium demonstrating stability over time at body temperature. The 3D printing technique described in this study may have applications in a variety of future 3D printed constructs.

## Abbreviations

3D: Three-dimensional
CT: Computed tomography
HU: Hounsfield unit
MRI: Magnetic resonance imaging
PCL: Polycaprolactone
ROI: Region of interest
